# Low-Dose Volumetric Modulated Arc Therapy for a Patient With Head and Neck Involvement of Mycosis Fungoides: A Case Report With a Review of Literature

**DOI:** 10.7759/cureus.26217

**Published:** 2022-06-22

**Authors:** Naoki Mukumoto, Haruo Inokuchi, Nobunari Hamaura, Mutsumi Yamagishi, Mai Sakagami, Shogo Matsuda, Daisuke Hayashi, Daisuke Tsuruta, Keiko Shibuya

**Affiliations:** 1 Department of Radiation Oncology, Graduate School of Medicine, Osaka Metropolitan University, Osaka, JPN; 2 Department of Dermatology, Graduate School of Medicine, Osaka Metropolitan University, Osaka, JPN

**Keywords:** lymphoma, vmat, total scalp irradiation, radiation, mycosis fungoides

## Abstract

Mycosis fungoides (MF) is the most common type of cutaneous T-cell lymphoma that slowly progresses over a period of years to decades. In some cases, lesions that spread to the scalp, neck, or facial skin can have a significant impact on cosmetic appearance and a patient's quality of life. Among the various treatments, radiation therapy is one of the most effective treatment modalities for patients with symptomatic cutaneous lesions. We report on an MF patient who had gradually increasing patches and plaques on the scalp, face, and neck and who underwent irradiation with 20 Gy administered in 10 fractions using volumetric modulated arc therapy. After undergoing this highly conformal technique, the patient obtained prolonged local control and significant alleviation of symptoms with acceptable adverse events. This technique constitutes a promising approach for treating a complex target due to its ability to provide homogeneous coverage of irregularly shaped target volumes along with its ability to preserve organs at risk. In addition, we systematically reviewed clinical reports on the management of extensive cutaneous lesions in MF patients undergoing other irradiation techniques.

## Introduction

Although mycosis fungoides (MF) is a rare disease with a relatively good prognosis when treated at an early stage, extensive cutaneous lesions can impair a patient's quality of life (QOL) [1-4]. Radiation therapy is one of the most effective treatment modalities for patients with all stages of MF, including symptomatic cutaneous lesions [5-7]. When skin lesions are solitary or few in number, local radiation therapy using electron or photon beam irradiation is generally indicated, while total skin electron beam therapy (TSEBT) is required when the skin lesions extend over the entire body [5-7]. However, when cutaneous lesions extend widely to areas that are uneven and with curving surfaces, such as the face and neck, these cases require advanced techniques of electron beam irradiation, which pose various challenges such as the heterogeneity of the target volume along with the long treatment time [8-10]. In order to achieve uniformity for the targeted lesions with complex shapes and for subjects with strict organ-at-risk dose constraints, new irradiation techniques have been developed. These new methodologies include intensity-modulated radiotherapy (IMRT) and volumetric modulated arc therapy (VMAT), with these techniques now used during daily clinical practice for cases of advanced head and neck cancer and brain tumors [11, 12]. This report presents data for a successful clinical treatment of extensive MF on the scalp, face, and neck using VMAT with 20 Gy administered in 10 fractions.

## Case presentation

An 82-year-old man presented with a tumor in the posterior auricular region and a three-year history of erythematous patches and plaques on the trunk, extremities, and face. A skin biopsy from a cutaneous chest lesion was performed and a histological evaluation of the specimen revealed the infiltration of atypical lymphocytes into the epidermis, which was positive for CD3, CD4, and CD5, and negative for CD79a, CD8, and CD20 (L26). Atypical lymphocytes were not seen in the peripheral blood. The serum level of the soluble interleukin-2 receptor was elevated at 848 U/mL, and anti-human T-cell leukemia virus 1 and 2 antibodies were not detected.

**Figure 1:**
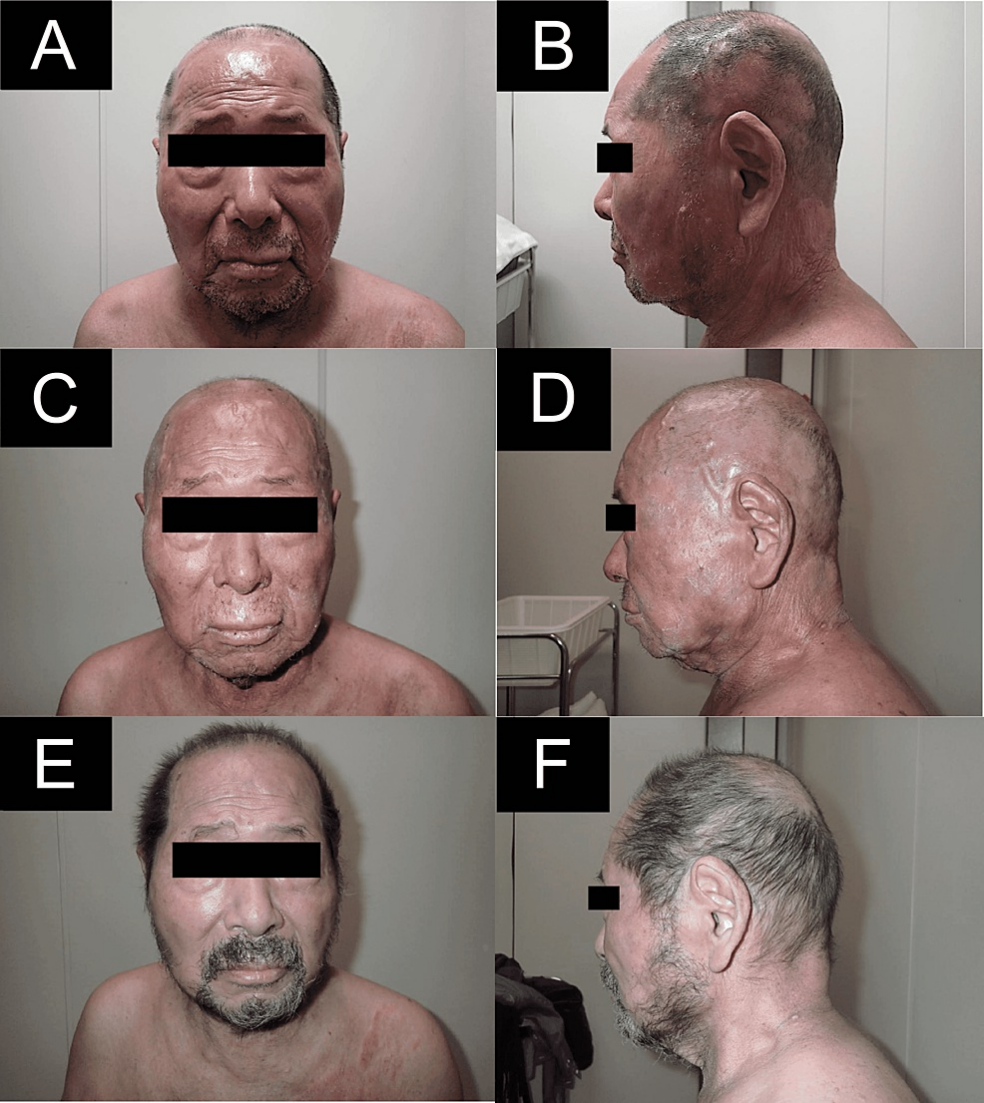
Clinical images Prior to the treatment, observed patches and plaques were found to have spread all over the scalp and on the skin of the face to the neck (A, B). Although at one week after treatment grade 1 radiation dermatitis, grade 2 alopecia, and mild dilation of the subcutaneous veins were observed, there was a tendency for the cutaneous lesions to be resolved (C, D). At four months after the treatment, there was complete response observed for the cutaneous lesions in addition to the resolution of the alopecia (E, F).

The patient was diagnosed with MF T3N0M0B0 Stage2B according to the International Society for Cutaneous Lymphomas/European Organization of Research and Treatment of Cancer (ISCL/EORTC) revised classification system. Although he was treated with chemotherapy (bexarotene) and skin-directed therapies such as topical agents and phototherapy, the cutaneous lesions became resistant to these therapies and the side effects such as renal impairment and hypertriglyceridemia made it difficult to administer adequate doses of chemotherapy. Patches and plaques gradually increased, most prominently on the facial skin and scalp, and this appearance of cutaneous lesions such as erythema and alopecia impaired the QOL of the patient (Figure 1A, 1B). The patient had no previous history of radiation therapy and was switched to radiation therapy using VMAT.

**Figure 2:**
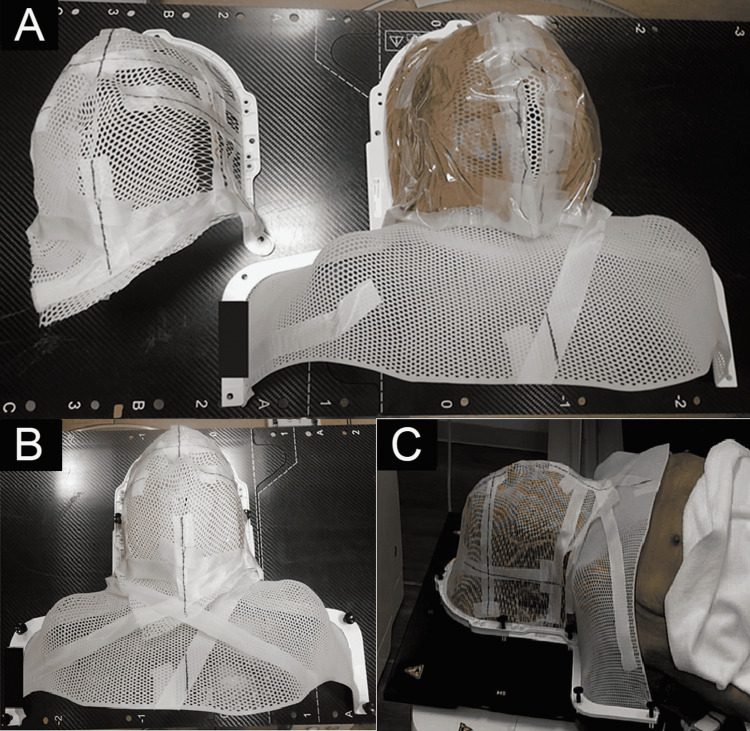
Patient immobilization device After being immobilized with a type S shell (Toyo Medic), a 0.5 cm bolus was placed to fit the patient, with a second type S shell then immobilized over the bolus (A, B). Patient positioning at CT simulation (C).

**Figure 3:**
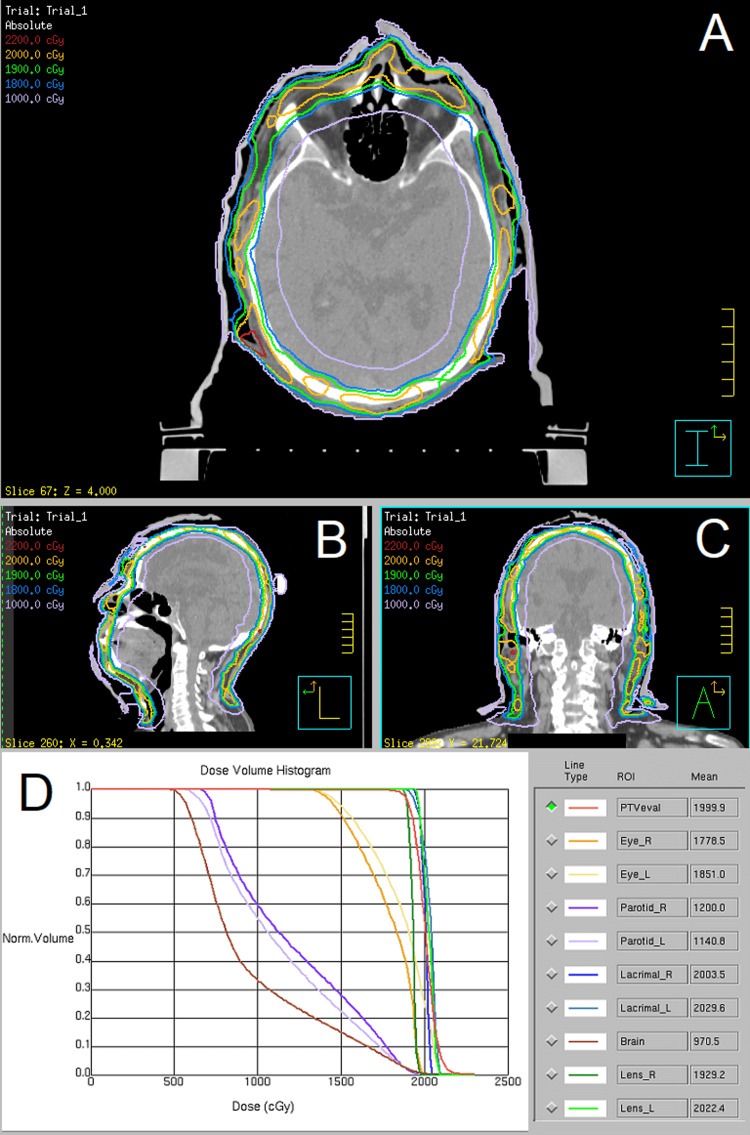
Volumetric modulated arc therapy plan Axial (A), sagittal (B), and coronal (C) view of the dose distribution. Dose-volume histogram and mean doses (cGy) of organs at risk (D).

Computed tomography (CT) simulation was performed in the supine position at a slice thickness of 2 mm. After immobilization of the subject using a type S shell (Toyo Medic), a 0.5 cm bolus was placed to fit the patient, with a second type S shell then immobilized over the bolus (Figure 2). VMAT plan was created using the Pinnacle treatment planning system version 9.10 (Philips). The clinical target volume (CTV) included a 4 mm area ranging from the skin surface of the entire scalp and from the face to the neck. The planning target volume (PTV) was defined as the CTV plus a 5 mm margin. The PTV was cropped by a 3 mm margin from the skin surface to form PTV evaluate (PTVeval) structure, which was used for dose normalization. A dose of 20 Gy in 10 fractions was prescribed to the mean dose of the PTVeval using a 6 MV photon beam from Versa HD (Elekta). VMAT plan was designed by 3 arc rotation fields to deliver a highly conformal dose distribution to the target with complex shapes, while minimizing the dose delivered to the organs at risk such as brain and parotid glands. Lenses and lacrimal glands were not spared due to eyelids involvement. Figure 3 shows the dose distribution and dose-volume histogram of the VMAT plan. The irradiation time was 555 seconds, with a total time starting from the time the patient entered the treatment room until leaving, of approximately 20 minutes. Toxicity evaluation was defined with the CTCAE v5 scale. Radiation therapy was performed in combination with the administration of low-dose bexarotene to control cutaneous lesions outside the irradiated area, with no adverse events observed during the treatment period. At one week after completion of the radiation therapy, the patient presented with grade 1 radiation dermatitis, grade 2 alopecia, and mild dilation of the subcutaneous veins, with cutaneous lesions observed to be less erythematous along with the absence of any bone marrow suppression (Figure 1C, 1D). Two months after completion of the radiation therapy, the cutaneous lesions had almost completely disappeared, although there was slight redness remaining in some areas. Four months after completion of the radiation therapy, complete response (CR) was observed for the cutaneous lesions and the patient’s normal hair had grown back (Figure 1E, 1F). At 16 months of completion of the radiation therapy, CR remained for the lesions within the irradiated area, and there were no observed late adverse events resulting from the administered irradiation.

## Discussion

MF is a peripheral T-cell lymphoma that primarily affects the skin and which is often treated using local electron beam irradiation or TSEBT [5-7]. However, with recent advances in irradiation techniques, the efficacy of total scalp irradiation using IMRT, VMAT, or total skin helical tomotherapy (TSHT) has been reported [13-17]. Moreover, these newer irradiation techniques have made it possible to deliver uniform doses to complexly shaped skin surfaces, thereby sparing the adjacent normal tissue and critical structures [14-17]. In addition, these treatments require less treatment time and can be performed in a supine position, which can help to reduce patient burden as compared to that associated with the use of complex electron beam irradiation.

Furthermore, some authors have reported good outcomes for cutaneous T-cell lymphoma of the scalp when using IMRT treatments, in conjunction with clinically acceptable adverse events [13-15]. While the efficacy of TSHT has also been reported in several cases, photon beams are more likely to cause severe bone marrow suppression as compared to electron beams due to the increased dose that is administered to the deeper parts of the body [16-19]. Death due to bone marrow suppression associated with TSHT has been previously reported [18].

Furthermore, it has also been reported that MF patients developed bone marrow suppression including grade 4 thrombocytopenia after TSHT despite receiving bone marrow mean doses (arms not included) that were as low as 1.66 and 2.3 Gy, respectively [19]. In our patient, although the lesion had spread to the trunk as well, we decided to only use VMAT to treat the skin on the head, face, and neck due to the consideration that irradiation to a wider area could potentially cause bone marrow suppression, with increases in the lesions on the scalp and face also reducing the QOL. Even at the previously reported lower dose of 20 Gy, we were able to obtain a relatively long-term local control, and furthermore, with the additional application of VMAT to the local lesions, there were minimal adverse events along with high patient satisfaction [5-7].

The advantages of treating our current case with VMAT were the shorter treatment times, the less laborious setup, and the high dose homogeneity to the target. These results suggest that this approach can be utilized for treating patients with other cutaneous malignancies that include the extensive spread of lesions on the scalp and face. 

## Conclusions

MF is a highly radiosensitive disease and radiation therapy plays an important role in the management of patients with all stages of MF. VMAT can deliver a highly conformal dose to the target with complex shapes, while minimizing the dose delivered to the organs at risk. For MF with extensive lesions that ranged over an area from the skin of the scalp to the face and neck, low-dose radiation therapy with VMAT was found to be very useful and led to excellent local control with little toxicity.
